# Georgia Medical & Surgical Encyclopedia

**Published:** 1860

**Authors:** 


### "Georgia Medical & Surgical Encyclopedia."

We have received the prospectus of a new Medical Journal, to be called the "Georgia Medical and Surgical Encyclopedia," and to be published in the town of Sandersville, Ga. The Journal is to be a monthly of forty-eight pages, and will be edited by Drs. Hollifield & Newsome; and furnished to subscribers at $2 a year in advance, or $2.50, if deferred until the expiration of the year. We will exchange with pleasure, and wish our friends all possible success — Address Drs. Hollifield & Newsome, Sandersville, Ga.

